# A comprehensive image dataset of American Sign Language hand gestures

**DOI:** 10.1016/j.dib.2026.112492

**Published:** 2026-01-20

**Authors:** Md. Famidul Islam Pranto, Md. Rifatul Islam, Md. Ali Akbor, Nabonita Ghosh, Md. Rahatun Alam, Sudipto Chaki, Md. Masudul Islam

**Affiliations:** Department of Computer Science and Engineering, Bangladesh University of Business and Technology, Dhaka 1216, Bangladesh

**Keywords:** American Sign Language, ASL hand gestures, Sign language recognition, Gesture dataset, Computer vision, Assistive technology

## Abstract

We present ASL-HG, a comprehensive American Sign Language (ASL) image dataset designed to advance gesture recognition and assistive technologies. The collection contains 36,000 static images across 36 classes, covering the full English alphabet (A–Z) and digits (0–9). Data were captured from 10 volunteers in Mirpur, Dhaka, Bangladesh, with each participant contributing 100 samples per class, ensuring a balanced distribution across subjects, genders, and skin tones. Unlike many existing ASL datasets, ASL-HG explicitly distinguishes between the letter “O” and the digit “0″ by including the standard two-handed ASL “zero” sign used in practical alphanumeric communication. The dataset is released in two complementary forms: raw images with natural indoor and outdoor backgrounds, and a MediaPipe-processed version with hand-segmented crops and predefined 80–20 train–test splits. This design supports both custom pre-processing and immediate model training. ASL-HG is intended to serve as a benchmark resource for developing robust and fair ASL recognition systems, reducing communication barriers for deaf and speech-impaired users, and enabling broader research in gesture-based human–computer interaction.

Specifications Table

**Table:** 

Subject	Computer Sciences
Specific subject area	Computer Vision, Pattern Recognition, Machine Learning, Deep Learning, Sign Language, Gesture Recognition, Assistive Technology
Type of data	JPG Image, Raw
Data collection	This dataset consists of 36,000 JPG images representing 36 American Sign Language (ASL) classes, covering the full English alphabet (A–Z) and digits (0–9). Images were contributed by 10 student volunteers residing in Mirpur, Dhaka, Bangladesh, during May–June 2025. Each participant provided 100 samples per class, resulting in a balanced distribution of 3600 images per individual. Static ASL hand gestures were captured using a smartphone camera in natural indoor and outdoor environments, with varied lighting, backgrounds, viewing angles, and distances, while ensuring clear visibility of the hand region. To improve usability, the dataset is released in two forms: the original raw images and a pre-processed version with MediaPipe-based hand segmentation and standardized train–test splits. This dual format supports both direct experimentation and efficient integration into computer vision pipelines. By incorporating balanced gender and skin tone representation along with explicit inclusion of the ASL sign for “zero,” which differs from the visually similar gesture for the letter “O,” the dataset prevents model confusion between the two classes and ensures accurate labelling of alphanumeric signs. This design provides a reliable and comprehensive foundation for sign recognition, assistive technology, and human–computer interaction research.
Data source location	The images were collected in Mirpur, Dhaka, Bangladesh (approximately 23.80° N, 90.36° E), with contributions from student volunteers residing in the area.
Data accessibility	Repository name: Mendeley Data Data identification number: https://doi.org/10.17632/j4y5w2c8w9.1 Direct URL to data: https://data.mendeley.com/datasets/j4y5w2c8w9/1 Instruction for accessing these data: Go to the download link and simply go to the ‘File’ tab and click download icon for downloading the data, or click the ‘Eye’ icon to view the dataset.

## 1 Value of the Data

• This dataset provides a high-quality and balanced collection of American Sign Language (ASL) hand gesture images, enabling researchers to build and evaluate robust gesture recognition models.
• It benefits the development of assistive technologies by supporting communication tools for the deaf and speech-impaired community, thereby promoting inclusivity.
• The dataset explicitly includes the standard ASL sign for the digit “0,” reducing the ambiguity between the gestures for “O” and “0” that is often present in other resources.
• Two complementary versions—raw images and a MediaPipe-processed version with predefined train–test splits—offer flexibility for both beginners and advanced machine learning practitioners.
• Beyond sign language recognition, the dataset can be reused for tasks such as hand detection, gesture-based human–computer interaction, pattern recognition, and computer vision model benchmarking.
• Diversity in participant gender and skin tone ensures broader representation, allowing researchers to develop fair and generalizable AI systems.

## 2 Background

American Sign Language (ASL) serves as a crucial communication medium for millions of deaf and speech-impaired individuals worldwide, enabling the visual expression of language, numbers, and emotions [1]. Despite its importance, the development of robust ASL recognition systems has been constrained by the limited availability of large, high-quality datasets that adequately represent both the English alphabet (A–Z) and numerical digits (0–9) [[9], [10], [11]]. Prior research has explored deep learning techniques for ASL recognition, including convolutional neural networks (CNNs) for static gestures [5,6], recurrent models such as LSTM and 3D-CNNs for dynamic gestures [2,3], attention-based graph networks [1], and hybrid multimodal frameworks combining multiple streams of input [4,7,8]. These studies have demonstrated promising results under controlled environments but are limited in real-world scenarios due to insufficient dataset diversity in lighting, hand orientation, backgrounds, and skin tones [[9], [10], [11]]. Recent Data in Brief contributions have highlighted the importance of well-documented, domain-specific datasets for advancing computer vision and pattern recognition research [[16], [17], [18], [19]]. In line with these efforts, ASL-HG provides a systematically collected and thoroughly described resource tailored to static American Sign Language hand gestures.

To address these limitations, the present work introduces a comprehensive ASL image dataset consisting of 36,000 high-resolution images across 36 classes. Images were collected from 10 volunteers under varied indoor and outdoor conditions, capturing diverse lighting, hand orientations, and natural backgrounds [12,13]. Each image has been pre-processed using MediaPipe for hand segmentation and organized into standardized train-test splits, enabling immediate integration into machine learning pipelines [14]. Special attention was given to avoid confusion between visually similar gestures, such as the letter “O” and the digit “0,” ensuring accurate labeling for alphanumeric recognition [12].

By providing both raw and processed versions, this dataset facilitates direct experimentation, serves as a benchmark for future ASL recognition research, and supports the development of inclusive human-computer interaction systems and assistive technologies for the deaf and speech-impaired community [1,12,14].

## 3 Data Description

The dataset, stored in the Mendeley Data repository [15], contains 36,000 JPG images across 36 classes representing the full English alphabet (A–Z) and digits (0–9). Data were collected in Mirpur, Dhaka, Bangladesh, during May–June 2025 from 10 student volunteers, with each participant contributing 100 images per class. The images capture hand gestures under proper lighting conditions and varied backgrounds, ensuring diversity in skin tone, hand shape, and angle. The dataset is provided in two forms: the original raw collection, which preserves natural variability, and a processed version in which MediaPipe was applied to segment the hands from the background, producing clean inputs suitable for model training. The processed set is split into training and testing subsets, with 80 % of the images (28,800) for training and 20 % (7200) for testing, while maintaining balanced representation across all classes. By offering both raw and processed versions, the dataset supports flexible use for computer vision and deep learning research, enabling custom preprocessing or direct model integration, and provides a standardized resource for sign language recognition and assistive technology development. Table 1 represents the dataset description and example images.

**Table 1:** ASL dataset gesture description.

SI.	Gesture	Class	Images	Description
1.		0	1000	ASL digit zero (palm version) - O-shaped hand placed against the palm of the other hand, used to distinguish the number zero from the letter O
2.		1	1000	ASL digit one - index finger extended upward, other fingers closed, static hand gesture
3.		2	1000	ASL digit two - index and middle fingers extended in V-shape, static hand gesture
4.		3	1000	ASL digit three - thumb, index, and middle fingers extended, static hand gesture
5.		4	1000	ASL digit four - four fingers extended upward, thumb folded in, static hand gesture
6.		5	1000	ASL digit five - all five fingers spread open and extended, static hand gesture
7.		6	1000	ASL digit six - thumb touches pinky finger, other three fingers extended, static hand gesture
8.		7	1000	ASL digit seven - thumb touches ring finger, other fingers extended, static hand gesture
9.		8	1000	ASL digit eight - thumb touches middle finger, other fingers extended, static hand gesture
10.		9	1000	ASL digit nine - thumb touches index finger forming a circle, other fingers extended, static hand gesture
11.		A	1000	ASL letter A - closed fist with thumb resting on the side of the hand, static hand gesture
12.		B	1000	ASL letter B - flat hand with all fingers together pointing up, thumb across palm, static hand gesture
13.		C	1000	ASL letter C - curved hand forming C-shape with thumb and fingers, static hand gesture
14.		D	1000	ASL letter D - index finger pointing up, thumb touches middle and ring fingers, static hand gesture
15.		E	1000	ASL letter E - fingers curled inward, touching thumb, forming a claw shape, static hand gesture
16.		F	1000	ASL letter F - thumb and index finger form a circle, the other three fingers extended up, static hand gesture
17.		G	1000	ASL letter G - index finger and thumb extended horizontally parallel to the ground, static hand gesture
18.		H	1000	ASL letter H - index and middle fingers extended horizontally together, static hand gesture
19.		I	1000	ASL letter I - pinky finger extended upward, other fingers closed with thumb across, static hand gesture
20.		J	1000	ASL letter J - pinky finger draws J-shape in air (captured static position), static hand gesture
21.		K	1000	ASL letter K - index and middle fingers extended in V-shape, thumb touches middle finger, static hand gesture
22.		L	1000	ASL letter L - index finger and thumb extended forming an l-shape, other fingers closed, static hand gesture
23.		M	1000	ASL letter M - thumb tucked under first three fingers, static hand gesture
24.		N	1000	ASL letter N - thumb tucked under first two fingers, static hand gesture
25.		O	1000	ASL letter O - all fingers and thumb form a circle or O-shape, static hand gesture
26.		P	1000	ASL letter P - similar to K but pointing downward at an angle, static hand gesture
27.		Q	1000	ASL letter Q - similar to G but pointing downward, thumb and index extended down, static hand gesture
28.		R	1000	ASL letter R - index and middle fingers crossed, other fingers closed, static hand gesture
29.		S	1000	ASL letter S - closed fist with thumb wrapped across front of fingers, static hand gesture
30.		T	1000	ASL letter T - thumb tucked between index and middle fingers, fist closed, static hand gesture
31.		U	1000	ASL letter U - index and middle fingers extended together upward, other fingers closed, static hand gesture
32.		V	1000	ASL letter V - index and middle fingers extended in V-shape, other fingers closed, static hand gesture
33.		W	1000	ASL letter W - index, middle, and ring fingers extended upward, pinky and thumb closed, static hand gesture
34.		X	1000	ASL letter X - index finger bent forming a hook shape, other fingers closed, static hand gesture
35.		Y	1000	ASL letter Y - thumb and pinky extended outward, other fingers closed, static hand gesture
36.		Z	1000	ASL letter Z - index finger traces Z-shape in air (captured static position), static hand gesture

## 4 Experimental Design, Materials and Methods

**Fig. 1:**
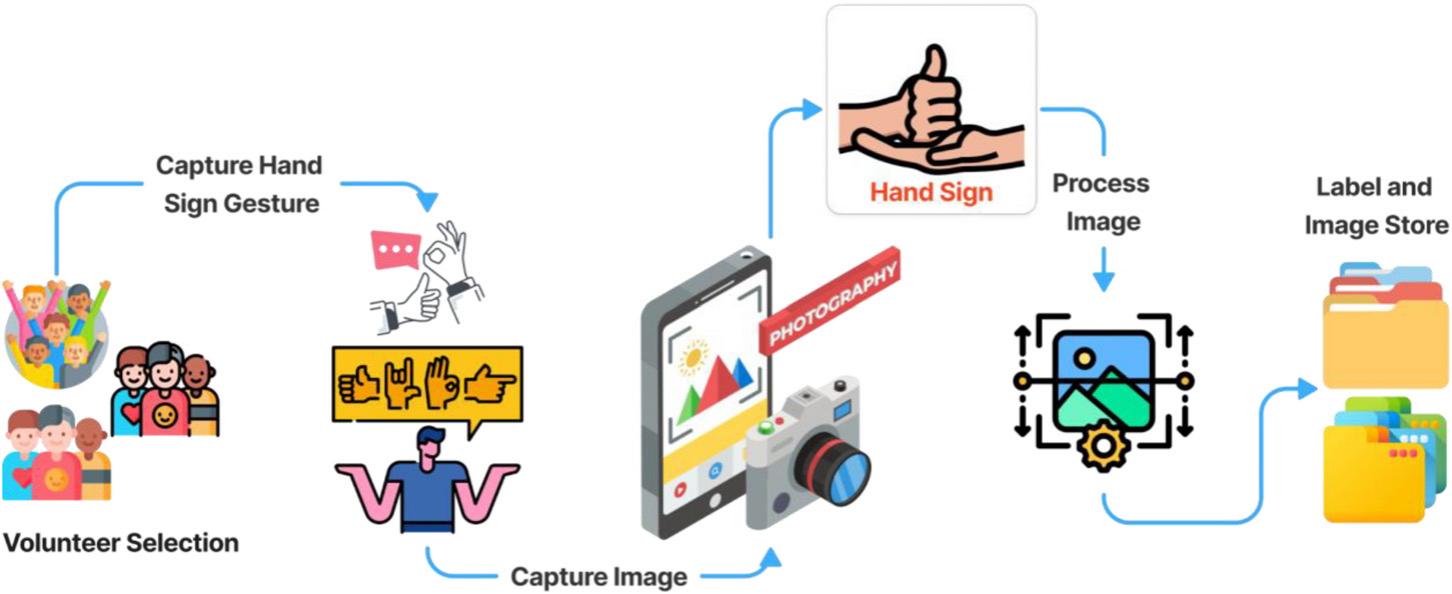
Dataset collection process step by step.

The image acquisition for each ASL class followed a systematic workflow designed to ensure diversity and representativeness across 36 gesture types, including A–Z and 0–9. Ten student volunteers contributed images, with 100 samples per class per participant, capturing natural variations in hand shapes, skin tones, and gesture execution. Images were taken in Mirpur, Dhaka, Bangladesh, during May–June 2025 using smartphone HD cameras under natural indoor and outdoor lighting conditions. To enhance dataset quality, gestures were reviewed to ensure clarity and consistency, while ambiguous images or those with excessive motion blur were excluded. The collected images were organized systematically by class, and a processed version was generated using MediaPipe for precise hand segmentation, removing background distractions and standardizing the hand region for machine learning and deep learning applications. Finally, the processed dataset was split into training and testing sets, maintaining an 80–20 distribution and balanced class representation, resulting in a structured, high-quality resource suitable for training robust computer vision and deep learning models for sign language recognition Fig. 1.

**Fig. 2:**
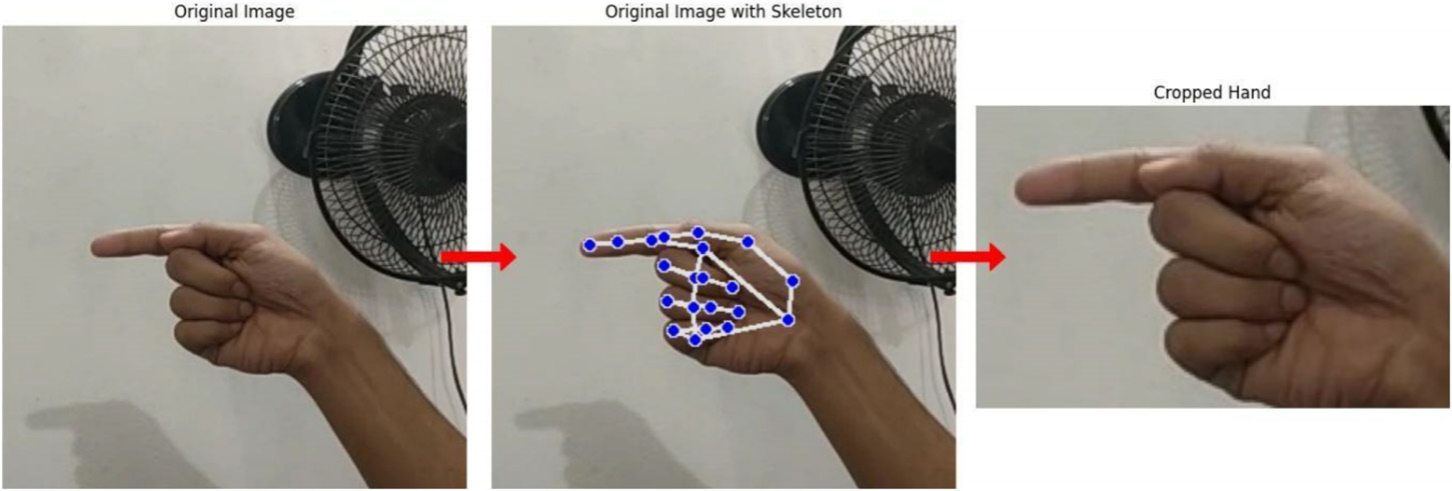
Hand gesture segmentation process using MediaPipe.

MediaPipe was employed in this study to preprocess images and enhance the quality and consistency of the ASL dataset. This framework automatically detects and crops hand regions from raw images, removing irrelevant background information and standardizing the input for model training. By focusing on the hand alone, the dataset ensures that models learn gesture-specific features rather than distractions from clothing, environment, or other objects. This preprocessing step improves model performance, generalizability, and interpretability. Fig. 2 illustrates example images before and after MediaPipe-based hand segmentation, demonstrating the effectiveness of this approach in creating cleaner and more informative inputs for ASL recognition models.

### 4.1 Camera Specification

All images were captured using an OPPO Reno5 (4 G) smartphone, equipped with a Snapdragon 720 G chipset, 8 GB RAM, and 128 GB internal storage. The rear camera setup includes a 64MP main wide lens, an 8MP ultra-wide lens, a 2MP macro lens, and a 2MP mono depth sensor. The front camera is 44MP, enabling high-resolution selfie captures. This setup ensured clear, detailed images of hand gestures suitable for machine learning, deep learning, and computer vision applications.

## Limitations

The dataset has several limitations. First, image collection was restricted to Mirpur, Dhaka, Bangladesh, which may limit geographic diversity and generalizability to other regions or populations. Second, although images were captured under varied natural lighting, inconsistencies in brightness, shadows, and background conditions may affect uniformity across the dataset. Third, while the dataset contains 36,000 images, deep learning models with extremely high complexity may still benefit from additional samples or data augmentation. Fourth, hand gestures were contributed by only 10 participants, so variations in hand size, shape, or subtle execution differences may not fully reflect the wider population. These factors may introduce biases related to geography, demographic representation, and imaging conditions, which should be considered when generalizing models trained on ASL-HG to broader populations or deployment environments. Nonetheless, by explicitly documenting these limitations, we provide a transparent basis for interpreting experimental results and a roadmap for future dataset extensions, including expanding to additional regions, increasing the number and diversity of participants, and incorporating more controlled imaging setups and dynamic gesture sequences.

## Ethics Statement

This study involved voluntary participation by individuals who contributed non-identifiable hand gesture images. No personal, sensitive, or health-related information was collected, and all data were fully anonymized prior to analysis. Participants were informed about the purpose and procedures of the study and provided informed consent before participation. According to institutional and national research guidelines, this type of anonymous, non-invasive data collection that does not involve personal identifiers does not require review or approval from an Institutional Review Board (IRB) or ethics committee. Therefore, ethical approval was not sought. The study adhered to the ethical principles of the Declaration of Helsinki and relevant institutional standards. No animal subjects were involved in this research.

## Consent to Participate Statement

All participants were informed about the purpose, scope, and procedures of the study before data collection. Participation was entirely voluntary, with no coercion or incentives. Because the data collected consisted only of non-identifiable hand gesture images and included no personal or sensitive information, written consent was not required under applicable institutional guidelines. However, informed verbal consent was obtained from all participants prior to participation. Participants were assured that the images would be used exclusively for research and academic publication purposes and that their identities would remain anonymous.

## Credit Author Statement

**Md. Famidul Islam Pranto**: conceptualization, data collection, software development, formal analysis, methodology, writing the original draft, and visualization. **Md. Rifatul Islam:** formal analysis, methodology, and validation. **Md. Ali Akbor:** formal analysis, methodology, and validation. **Nabonita Ghosh**: data curation, investigation. **Md. Rahatun Alam:** data curation, investigation. **Sudipto Chaki:** supervision, investigation, writing review, and editing. **Md. Masudul Islam:** supervision, investigation, writing review, and editing.

## Declaration of Competing Interest

The authors declare that they have no known competing financial interests or personal relationships that could have appeared to influence the work reported in this paper.

## Data Availability

Mendeley DataASL-HG: American Sign Language Hand Gesture Image Dataset (Original data)
